# Identifying Risk Factors Associated with Fatal Drowning Accidents in the Paediatric Population: A Review of International Evidence

**DOI:** 10.7759/cureus.6201

**Published:** 2019-11-19

**Authors:** Martin Davey, Sean Callinan, Leona Nertney

**Affiliations:** 1 Trauma and Orthopaedics, Royal College of Surgeons, Dublin, IRL; 2 Paediatrics, Royal College of Surgeons, Dublin, IRL

**Keywords:** swimming, swim, drowning, pediatrics, paediatrics, swim ability, guidelines, risk factors, risk reduction

## Abstract

Introduction: Evidence from Ireland’s Childhood Mortality Register demonstrates that drowning is the second leading cause of death in children. It occurs more commonly in adolescent males engaged in summer water-based activities and in children aged 1-4 years with access to swimming pools/unprotected water sources. Despite being an island nation, a significant lack of guidelines exists to reduce drowning accidents in these at-risk populations.

Aim: Review international evidence surrounding risk factors associated with drowning accidents in the paediatric population and existing risk-reduction guidelines.

Methods: Structured review of Cochrane, Cinahl, Pubmed Web of Science databases performed using search terms: (“risk factors” AND “drowning”), (“risk reduction” OR “prevention” OR “swim ability” AND “drowning”). Studies were included if satisfied age criteria (0-18 years).

Results: Evidence suggests that boys are at highest risk of drowning (1-4 yrs in swimming pools; adolescents in freshwater) with inadequate surveillance, inadequate availability of first responders, certain clinical diagnoses (developmental delay and seizure disorders), lack of swimming ability, and substance misuse in adolescents all posing an increased risk. Formal swimming education in those aged 4+ years, training of supervising adults in safe rescue, installation of isolation barriers, enforcing water safety guidelines, and regulations are all recommended by International Advisory Groups for prevention of drowning.

Conclusion: In Ireland, drowning is the second leading cause of accidental paediatric death in the post-neonatal period, and an important cause of childhood fatalities globally. Risk factors increasing the likelihood of fatal paediatric drownings include gender and distinct age peaks. Certain modifiable risk factors relate to peri-event factors such as lack of supervision, to post-event responses, in particular including lack of trained personnel at the scene. There is a poverty of guidelines specifically targeting the paediatric populations; guidelines generally tend to be included into adult drowning reduction strategies. Specific targeting is required to protect those most at risk.

## Introduction and background

In both paediatrics and adult medicine, drowning is a leading cause of injury-related death. In recent years, the incidence of fatal drowning, as well as out-of-hospital cardiac arrest, resulting in death have been increasing [1-3]. Many definitions of ‘drowning’ exist concurrently; it is broadly defined as “death within 24 hours from suffocation by submersion in a liquid, normally fresh or seawater”. This is contrasting in definition to ‘near-drowning’, which is classified as “survival for more than 24 hours (even if temporary) from suffocation by submersion”[4]. These definitions emphasize the survival time post-submersion (if any), as opposed to the quality of life, morbidity or mortality experienced by the victim. Drowning, similar to burns (8%), is estimated to be the second most common cause of accidental paediatric death (8%), after road traffic accidents (65%) [5].

This review will explore potential risk factors which may increase the likelihood of a fatal drowning episode occurring in the paediatric age group. A review of current international guidelines targeting drowning reduction strategies is included.

## Review

Methods

**Figure 1:**
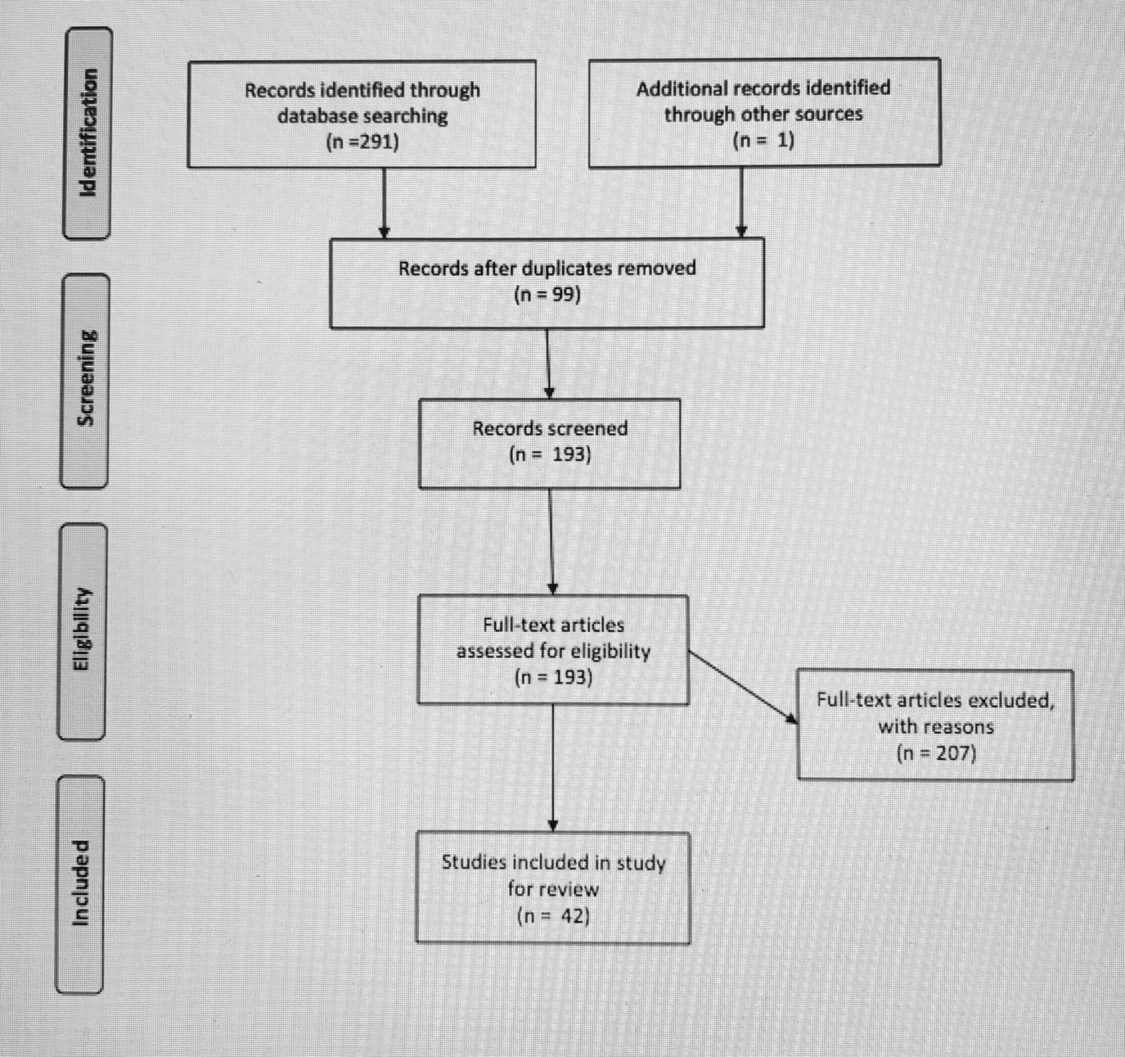
Preferred Reporting Items for Systematic Reviews and Meta-Analysis (PRISMA) Guidelines

A structured review of Cochrane, Cinahl, Pubmed Web of Science databases was performed using search terms: (“risk factors” AND “drowning”), (“risk reduction” OR “prevention” OR “swim ability” AND “drowning”). Studies were included if satisfied age criteria (0-18 years). This is further illustrated in Figure 1.

Risk factors associated with fatal paediatric drowning events

Environment

Inadequate surveillance is the most common reason for infant and toddler drownings; supervising adults should be within touching distance of children at all times to reduce the risk of drowning [2]. There is an increased risk of death from drowning in cases of poor immediate response to the incident; i.e. less cardiopulmonary resuscitation bystanders/lower availability of support by life-guards [2].

Co-morbidities

The presence of co-morbidities, namely epilepsy, genetic syndromes resulting in developmental delay and autism spectrum disorder places the child at an increased risk of drowning [6].

Demographics

Males are at higher risk of drowning across all age groups; with peak incidence seen in boys aged 1-4 (deaths occurring predominantly in swimming pools) and adolescent males (deaths occurring predominantly in freshwater) [1]. There are higher levels of paediatric deaths secondary to drowning in developing countries, which could be contributed to a number of confounders or a global poorer standard of medical care in these locations [7].

Substance Abuse

In the adolescent group, incidence of accidental death has been found to be higher in those victims who had alcohol or illicit substance prior to or whilst in the water (30%-50% of cases) [7].

Swimming ability of children

Swimming ability is a major factor in paediatric water difficulty [2]. Paediatric deaths from drowning in the cohort aged four years or younger has been principally attributed that based on their gross motor skills, these victims are not developmentally able to swim [1]. Similarly, children are noted to be less likely to drown having participated in swimming lessons when developmentally able (i.e. aged 4+) [7]. Therefore, children should be taught to swim as soon as developmentally possible. In those aged 4+, there is increased likelihood of drowning if unable to play in water up to head level or swim continuously for 60 seconds [7].

It is important to identify the swimming ability of children and their readiness to deal with difficult circumstances in water situations. If there is currently no area for improvement then an intervention will result in little effect. Conversely, it may be quite apparent that there are limitations in the ability of children to swim competently, which contributes to a significant cause and proportion of paediatric drowning incidences. Therefore there may be a significant need for increased level of swimming lessons and education on water safety for adults and children alike. Guidelines can be non-specific and apply to all ages and all engagements with water sources or they may be specific to certain population subsets and situations. Brenner et al. reported the results of their case-controlled retrospective study which found that children under the age of five who died due to drowning were less likely to have received training compared to control cases [7].

It is difficult to assess the swim ability of a specific population and there will be significant variations due to specific circumstances such as age, level of fitness, development, and socioeconomic status. In 1986, Langley & Silva stated in a cohort study of 9-year-old children in New Zealand that 29% “could swim only a few strokes or not at all” [8]. Furthermore, there was a disproportionate amount of children with superior swimming ability who came from high socio-economic backgrounds demonstrating an inequality based on social background. Irwin et al. (2009) conducted a survey and identified that poor minority children report that they feel more “at risk” with regard to their swimming ability when compared to Caucasian children. Swimming ability was also negatively effected by race being reported as worse by African-American and Latino-Hispanic [9].

It has been documented in a cohort of 109 children aged 24-42 months that their swimming ability and safety behavior improved from baseline after eight or 12 weeks of training (Asher et al. 1995) [10]. The training included lessons on swimming and safety skills only twice a week and included three skill sets including: “out-of-water safety behavior (deck behaviour); swimming ability; in-water safety skills.” The group, which received 12 weeks of lessons, had superior skills to the eight-week training group, representing an ongoing level of mastery of new learnt skills. This study demonstrated that constant supervision and barriers to water access remain of paramount importance in children at this age in order to improved swim ability and overall water safety behaviour.

In 2015, a study by Quan et al. aimed to outline a standardized definition of ‘Water Competency’ for the American Red Cross. They stated that children should attain a certain level of swimming ability before being allowed to engage with the deep end of a pool [11]. They cite Stallman, et al. (2008) who says that it is mandatory in Norway for students to complete a 200-metre swim test [12]. They attempted to form a definition for Water Competency by surveying a number of experts in order to discover what should be required of children to demonstrate their competency of swimming. They recommend a variety of skills including being able to swim a certain distance, floating or treading water, specific strokes and understandably a very important aspect of water safety entry and exit skills. Quan stated that there is no predetermined set of standards, which can be applied to all who engage with water and they should be organization specific [11]. The same study reported that there are a number of skills that must be mastered prior to a child having full swim ability; these are outlined in Table 1.

**Table 1:** Skills Required

Skills Required
Distance swims 10-75 m
Floating/treading water 10-300s, 30-60s
Perform swim strokes – on front or back
Entry + / or exit skills - some required jumping
Duration swimming – 1-5 min
Swimwear – required to do in street clothes
Breath control skills – required face submersion
Demonstrate rescue techniques – reach or throw
Perform under different water conditions – river vs pool.

Swimming performance tests could prove beneficial in identifying weak swimmers and those at risk of drowning due to poor technique and those who struggle in the water due to inadequate fitness. Therefore the test could function as a fitness screening and safety screening with a view to improve both which would possibly augment both aspects symbiotically. Quan et al. stated the importance of considering the specific environment and conditions, which apply to a given situation [11]. Each and every skill recommended in this publication should be applied to the environment it is intended for. The proposed American Red Cross definition for Water Competency has been outlined in Table 2.

**Table 2:** American Red Cross Water Competencies

American Red Cross Water Competencies
Entry with total submersion
Recovery to surface and thread water for at least one minute
Change in body orientation allowing repositioning, turning at least 180 degrees
Propulsion including leveling off and moving for at least 25 meters.
Exit from water

The definition is essentially a functional swimming test and does not include any mention of attitude, behaviour, or planning. These are important aspects to consider when recommending a definition on the level of competency one should have when engaging with water to ensure the safety of themselves and others. They note their own limitations in solely focusing on the motor skill elements of swimming ability.

Swimming skills and safety instructions for parents should both be incorporated into swimming programs for infants and toddlers (The Committee on Sports Medicine and Fitness and Committee on Injury and Poison Prevention, 2000). They importantly state that despite the quality of the program it is not possible to instruct young children to a point that they understand the dangers of drowning. Furthermore, they reiterate that safety programs have not yet adequately demonstrated a resultant decrease in drowning rates and parents must not consider them to operate as certainty of safety against drowning. As a result of this supervision is paramount at all times but in particular for young children. The paper again brings up the point that swimming skills are separate from water safety skills, which include: “survival flotation, energy conservation “swimming,” or poolside behaviour”. Cited in their paper, McGraw (1939) notes that children exhibit basic swimming skills such as paddling at the age of one [3].

This could represent that the natural neuromuscular developmental patterns enable survival when adequate time and exposure are given. However when a child is placed in a situation out of proportion to their physical or technical ability they are compromised. Furthermore, this doesn’t account for the behaviour of children, which as they grow older becomes more explorative and disinhibited particularly when with friends. There hasn’t been a definitive recommendation with regards to what age or accomplished developmental milestone are most appropriate to begin swimming training at [3]. Blanksby et al. (1995) noted that it took 5-year-olds a shorter duration to achieve a satisfactory level of swimming skill, in the form of swimming a 10 m front crawl, when compared with younger ages [13].

Current guidelines

There are currently no accepted gold-standard guidelines in modern literature explicitly aimed at identifying risk factors and prevention of accidental drowning specific to the paediatric age group. There are numerous advisory sources on preventative measures for both adults and children, with much of the focus being on children in the 1-4 age group.

In January 2016, The National Institute of Clinical Excellence (‘NICE’) outlined “Preventing unintentional injury in under 15s” but this is a non-specific set of guidelines aimed at child protection agencies in the interest of protecting vulnerable children in the U.K from ‘unintentional injury’ or drowning. No similar paper has been published by NICE in hope of preventing drowning specifically in the paediatric cohort [14].

Many bodies have outlined preventative measures of drowning for the general public; however few are specifically in reference solely to the paediatric population. The ‘American Academy of Pediatrics’ (AAP) outlined such measures in 2010 in their paper titled “Policy Statement- Prevention of Drowning” [1]. The position of the AAP is that children are not developmentally ready for swimming lessons until after their fourth birthday. This motor development milestone (age four) includes the necessary skills to swim adequately and safely under supervision of a trained bystander. The AAP appreciate that there is no accepted evidence to suggest that there is lower rates of drowning in infants and toddlers who have attended aquatic programmes for those under the age of four (due to proclaimed less ‘fear of water’) as many studies show higher rates in the same demographic. However, all children should be taught to swim when developmentally able (on average; aged four and over) [1].

The AAP outline that supervision of all young children around any water is a paramount preventative measure in ensuring their safety and reducing drowning rates. Adequate swimming skills by the supervising adult are crucial in case of an emergency, with “touch supervision” being necessary with younger children [1]. Any child with seizure disorders should be supervised at all times when in any body of water (even in baths), regardless of age or swimming ability [1,6]. Parents, caregivers, and pool owners should learn CPR and keep a telephone close by in case of an unforeseen pool event to reduce risk of drowning.

The same organization also outlined that there is strong evidence to suggest that installation of four-sided fencing which completely encloses the pool is effective in preventing more than 50% of all drowning incidents in young children. Similarly, the AAP states that evidence is severely lacking to suggest that pool alarms or pool covers are an adequately safe replacement for pool fencing in preventing paediatric drowning.

The AAP also made preventative recommendations for those choosing to use outdoor freshwater. When selecting an open body of water for swimming with children of any age, a site with lifeguards should be prioritized. Walking, skating or riding on weak or thawing ice on a body of water should be avoided to reduce the risk of drowning incidents.

In 2017, the World Health Organization’s (WHO) guidelines titled “Preventing drowning: an implementation guide”, a series of recommendations to prevent drowning in adults and children have been established [15]. These include six interventions to prevent drowning at each potential drowning site (many of which have been outlined above) as well as four community & national strategies aimed at those living in the developing world [15]. These six interventions include: providing safe places away from water for pre-school children, installing barriers controlling access to water, teaching school children (aged over six years) swimming and water safety skills, building resilience to manage flood risks, training bystanders in safe rescue and resuscitation and enforcing safe boating, shipping and ferry regulations.

Four strategies to support drowning prevention interventions were also outlined: promoting multi-sectoral collaborations, strengthening public awareness of drowning through strategic communications, establishing a water safety plan and carrying out applicable research [15].

The WHO also identified that children in developing countries are at an increased risk of drowning globally, especially when situated in island countries such as those in the Pacific Islands and South-East Asia. Boys were also identified to be a higher risk group than girls, and drowning emergencies were substantially more common in children aged 6 years and under, as well as those who had never attended swimming lessons [15].

In 2009, a paper published by Chandy et al. titled “Drowning (submersion injuries)”, similar prevention strategies were outlined, including secure fencing and gating of swimming pools [16]. This paper suggested that measures can eliminate virtually all drowning in children under the age of four and 80% of total swimming pool drowning amongst paediatrics and adults alike. Guidance was given to parents and caregivers to supervise children in all bodies of water, including shallow areas, including but not limited to toilets, sinks and even buckets of water [16]. As this paper was based on drowning all age groups, it also recommended never swimming alone, use of floatation devices and avoidance of alcohol and illicit drugs; these recommendations are applicable to older children and teenagers alike [16].

Moran et al. (2011) discuss safety in all situations involving water as they highlight the significance of open water recreational activities and drowning incidences [17]. The International Task Force on Open Water Drowning Prevention (ITFDP) was formed and published 16 generic open water messages. A particular emphasis was placed on the importance of supervision and avoidance of alcohol use in adolescents as it is supported with strong evidence. With regards to swimming ability they state that: “Being able to swim reduces the chance of a serious incident but does not guarantee safety” and: “Water safety is more than just having swimming skills. It is also having the confidence, knowledge, skills, and attitudes to be safe in and around water” [17].

They discuss that it would be advantageous to exhibit adequate swimming ability with skills such as being able to tread water, swim underwater, and breathe correctly. However, they contrast that there are no studies, which demonstrate that these improved skills learnt through swimming lessons would lower drowning rates prospectively. Furthermore, they prudently discuss the risks of providing swimming lessons to children adolescents, which include providing false confidence of ability and increasing exposure to dangerous situations when not yet prepared.

Recommendations

Despite the current literature being relatively thorough and helpful in it’s own right, it does not currently suggest adequate, specific guidelines to reduce the risk of drowning in children. One clinically relevant and specific set of gold standard guidelines for parents and caregivers has yet to be established and accepted in prevention and risk reduction of drowning episodes in the paediatric population. Further research must be conducted to evaluate the current swimming ability of children applied to the environment which the plan to participate at. Swimming lessons in Ireland at present are privately-funded, and in order to improve safety outcomes across all socioeconomic groups would need to be government-funded or subsidised. Swimming programs could be instituted not only as a means of improving safety outcomes but also to increase fitness levels of children. Parent supervision must be strongly emphasized at all times; in addition to this parents and guardians should be encouraged to receive training for dealing with emergency situations and the performance of cardiopulmonary resuscitation. In order to increase the likelihood of such developments in future, a national training scheme for all water safety including the prevention of drowning in high-risk groups such as the paediatric population should be recommended.

## Conclusions

In Ireland, drowning is the second leading cause of accidental paediatric death in the post-neonatal period, and an important cause of childhood fatalities globally. As discussed, risk factors increasing the likelihood of fatal paediatric drownings include gender and distinct age peaks. Certain modifiable risk factors relate to peri-event factors such as lack of supervision, to post-event responses, in particular including lack of trained personnel at the scene. There is a poverty of guidelines specifically targeting the paediatric populations; guidelines generally tend to be included into adult drowning reduction strategies. Specific targeting is required to protect those most at risk.
